# MicroRNA-34a regulates WNT/TCF7 signaling and inhibits bone metastasis in Ras-activated prostate cancer

**DOI:** 10.18632/oncotarget.2690

**Published:** 2014-11-25

**Authors:** Wei-Yu Chen, Shih-Yang Liu, Yung-Sheng Chang, Juan Juan Yin, Hsiu-lien Yeh, Tarek H. Mouhieddine, Ola Hadadeh, Wassim Abou-Kheir, Yen-Nien Liu

**Affiliations:** ^1^ Department of Pathology, Wan Fang Hospital, Taipei Medical University, Taipei, Taiwan; ^2^ Department of Pathology, School of Medicine, College of Medicine, Taipei Medical University, Taipei, Taiwan; ^3^ Department of Acupuncture and Manipulation, College of International Education, Tianjin University of Traditional Chinese Medicine, Tianjin, China; ^4^ Graduate Institute of Cancer Biology and Drug Discovery, College of Medical Science and Technology, Taipei Medical University, Taipei, Taiwan; ^5^ Cell and Cancer Biology Branch, National Cancer Institute, National Institutes of Health, Bethesda, MD, USA; ^6^ Institute of Information System and Applications, National Tsing Hua University, HsinChu, Taiwan; ^7^ Department of Anatomy, Cell Biology and Physiological Sciences, Faculty of Medicine, American University of Beirut, Beirut, Lebanon

**Keywords:** Prostate cancer, bone metastasis, miR-34a, TCF7, BIRC5

## Abstract

Aberrant activation of Ras and WNT signaling are key events that have been shown to be up-regulated in prostate cancer that has metastasized to the bone. However, the regulatory mechanism of combinatorial Ras and WNT signaling in advanced prostate cancer is still unclear. *TCF7*, a WNT signaling-related gene, has been implicated as a critical factor in bone metastasis, and here we show that *TCF7* is a direct target of miR-34a. In samples of prostate cancer patients, miR-34a levels are inversely correlated with *TCF7* expression and a WNT dependent gene signature. Ectopic miR-34a expression inhibited bone metastasis and reduced cancer cell proliferation in a Ras-dependent xenograft model. We demonstrate that miR-34a can directly interfere with the gene expression of the anti-proliferative *BIRC5*, by targeting *BIRC5* 3′UTR. Importantly, BIRC5 overexpression was sufficient to reconstitute anti-apoptotic signaling in cells expressing high levels of miR-34a. In prostate cancer patients, we found that *BIRC5* levels were positively correlated with a Ras signaling signature expression. Our data show that the bone metastasis and anti-apoptotic effects found in Ras signaling-activated prostate cancer cells require miR-34a deficiency, which in turn aids in cell survival by activating the WNT and anti-apoptotic signaling pathways thereby inducing TCF7 and BIRC5 expressions.

## INTRODUCTION

Advanced prostate cancer is mostly associated with metastasis, typically to the bones, causing both osteoblastic and osteolytic lesions [1, 2]. However, the mechanisms of selective metastasis of prostate cancer cells to the bones remain unknown. It is believed that there are unique molecular signatures that predispose a prostate cancer patient to metastasis. Activation of the Ras signaling pathway is among the most widely observed phenomenon during metastasis in advanced prostate cancer [3, 4], and various lines of evidence suggest that Ras-dependent signaling contributes to the aggressiveness of advanced prostate cancer [5, 6]. Moreover, up-regulation of Ras-mediated signaling cascades may reflect up-regulations in various pathways such as the mitogen-activated protein kinase (MAPK), phosphatidylinositol 3-kinase (PI3K)/AKT cascades [7, 8], and WNT signaling [9]. A direct synergy between Ras and WNT signaling cascades are emerging as key events driving prostate cancer progression into invasive carcinoma by up-regulating genes such as cyclooxygenase-2 [10] and c-Myc [11, 12], indicating the critical convergence of these two pathways.

WNT signaling is a critical pathway that proceeds through a number of different genetic defects before developing prostate cancer [13]. The development of cancer via aberrant WNT signaling most likely results from stabilized β-catenin which in turn mediates inappropriate gene activation [14]. Accumulated cytoplasmic β-catenin translocates to the nucleus where it associates with members of the T-cell factor (TCF) and lymphoid enhancer factor (LEF). The β-catenin-TCF/LEF complex activates the transcription of target genes including c-Myc [11], c-jun, the metalloproteinase matrilysin [15], and cyclin D1 [16]. A study of the possible underlying mechanism indicated that these effects occurred through silencing of the WNT signaling pathway [17]. This was apparent by the loss of the nuclear accumulation of β-catenin and decreased transcriptional activity of transcription factor 7 (TCF7), suggesting that TCF7 may play a key role in promoting the oncogenic activity of the WNT signaling pathway [17]. However, additional defects in the regulation of the WNT signaling that contribute to tumor progression are yet to be discovered.

Many molecular regulators, such as microRNAs (miRs), play a significant role in regulating the WNT-β-catenin pathway [18]. miRs alter the expression of canonical WNT cascade genes by binding to the 3′UTR region of the target mRNA. This usually results in translational repression or mRNA degradation, which affects early embryonic development as well as tumorigenesis and metastasis [19, 20]. For example, the silencing of miR-34a and subsequent up-regulation of its target, β-catenin, was associated with liver metastases of colon cancer [21]. In support of prostate cancer tumorigenesis, the microRNA-34 (miR-34) family has shown anti-proliferative and apoptotic roles and its inactivation has been reported in malignant prostate cancer [22, 23]. miR-34 functions as a tumor suppressor and, thus, inducing miR-34a expression in human prostate cancer PC-3 cells inhibits *in vitro* cell proliferation and invasion and promotes apoptosis [24]. Recent studies have demonstrated that miR-34a modulates the canonical WNT cascade in breast cancer [20], however, the ability of miR-34a in modulating the WNT and Ras pathways in prostate cancer remains largely elusive.

The presence of Ras mutations as a cause of resistance to apoptosis in various cancers brought a major challenge in the treatment of metastasis [25]. Accumulating evidence shows that cancer's anti-apoptotic ability is a hallmark of cancer and is typically potentiated by a small number of anti-apoptotic proteins [26, 27]. The most studied proteins are the anti-apoptotic BCL-2 family members, inhibitors of apoptosis proteins, and caspase inhibitors [28, 29]. Although the intrinsic molecular mechanisms of evading apoptosis in cancer remain largely unknown, a wealth of biochemical and genetic studies indicates that Ras proteins control a complex molecular circuitry that affects multiple cellular processes that drive tumorigenesis [30–32]. We investigated the regulatory mechanisms by which miR-34a targets the WNT cascade and anti-apoptotic signaling. We also showed that miR-34a overexpression contributes to the induction of apoptosis in Ras-activated prostate cancer cells. In this paper, we demonstrate a direct link between the loss of miR-34a and activation of the canonical WNT signaling and anti-apoptotic pathways, and we further explored the therapeutic role of miR-34a in being a diagnostic marker in Ras-dependent prostate cancer patients.

## RESULTS

### Identification of miR-34a as a metastasis-inhibiting miR in Ras-activated prostate cancer

**Figure 1:**
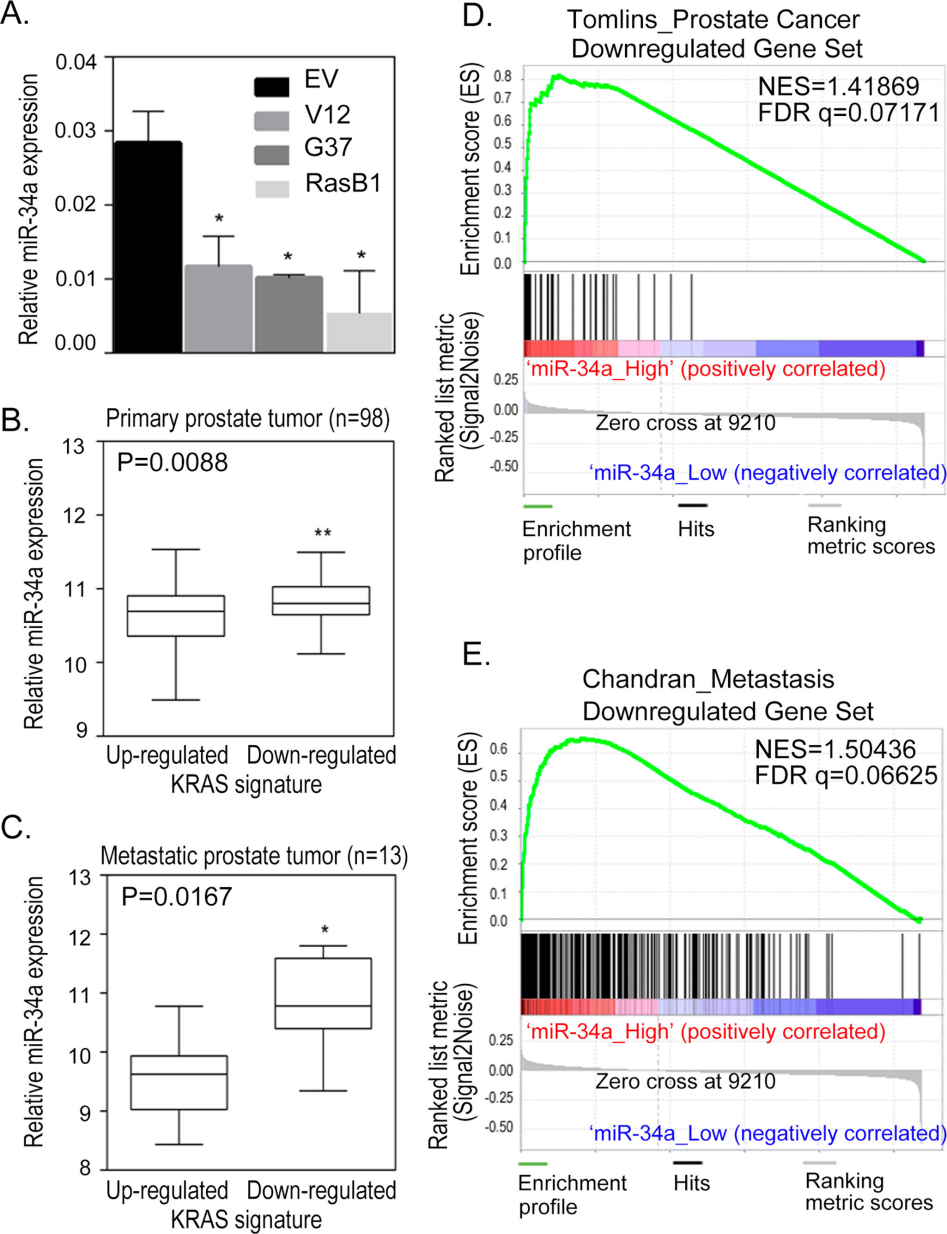
Reduction in miR-34a expression is related to Ras-induced prostate cancer metastasis **(A)** qRT-PCR of miR-34a expression levels determined in DU145 cells with an empty vector (EV), RasV12 (V12) or RasG37 (G37 and RasB1) mutant. miRNA expression was normalized to *SNORD48*. Data represent means ± SEM, *n* = 3. *: vs. EV. *p < 0.05. (**B** and **C**) Relative miR-34a expression in the human prostate carcinoma dataset segregated into up- and down- regulated KRAS signature in tissues of primary (B) and metastatic (C) stage prostate cancer. **(D)** Gene set enrichment analysis (GSEA) showing enrichment of Taylor prostate cancer dataset of down-regulated prostate cancer responsive gene signatures in prostate cancer highly expressing miR-34a. **(E)** GSEA showing enrichment of Taylor prostate cancer dataset of down-regulated metastatic prostate cancer responsive gene signatures in prostate cancer highly expressing miR-34a. False discovery rate (FDR), normalized enrichment score (NES).

To study the genes involved in Ras-driven prostate cancer metastasis, we chose a previously described model of human prostate cancer which utilizes DU145 cells infected with a lentiviral K-Ras mutation construct: RasV12G37 [33]. Following mouse intra-cardiac and orthotopic prostate injections, the DU145/RasV12G37 (G37) cell line displayed a dramatic increase in bone and brain metastasis within one month only [33]. The cell line used in this paper, DU145/RasB1 (RasB1), was isolated from a prostate tumor that has metastasized to the bone [34]. This cell line metastasizes to the bone in 2–4 weeks with a high frequency and provides a reliable and reproducible model to study the molecular mechanism of bone metastasis. It has been shown that miR-34a expression is down-regulated in patients with prostate cancer compared to people with normal prostate tissue [24]. We sought to determine whether miR-34a has a role in tumor progression in Ras signaling-activated prostate cancer cells, and found that the highly metastatic human prostate cancer cell line DU145/RasV12 (V12) [33], G37 or RasB1 (Supplementary Table S1) have reduced miR-34a expression (Figure 1A). In addition, human prostate tumor samples showed a significant reduction in miR34a expression compared to normal prostate tissues (Supplementary Figure S1A). We extended our analysis to a publicly available prostate data set on 99 primary tumors and 13 distant metastasis tissue specimens collected and analyzed at Memorial Sloan-Kettering Cancer Center (MSKCC) [6]. We divided the specimens into two groups of up- and down-regulated KRAS signaling gene expression signatures based on a measure of relative mRNA expression. An analysis of mean expression confirmed that miR-34a was highly expressed in tissues of primary (Figure 1B) and metastatic (Figure 1C) stage prostate cancer with down-regulated KRAS signatures. These data provide information regarding potential crosstalk within the Ras signaling pathway, downstream of miR-34a. Furthermore, we tested the relationship between miR-34a and prostate cancer progression via a gene set enrichment analysis (GSEA) and observed a significant increase in prostate cancer metastasis-inhibiting gene signatures in samples with high miR-34a expression (Figures 1D and 1E, and Supplementary Figure S1B). In summary, our results support the idea that the miR-34a expression is a downstream event of the Ras signaling pathway and involved in prostate cancer metastasis.

### Loss of miR-34a is associated with activated WNT signaling

**Figure 2:**
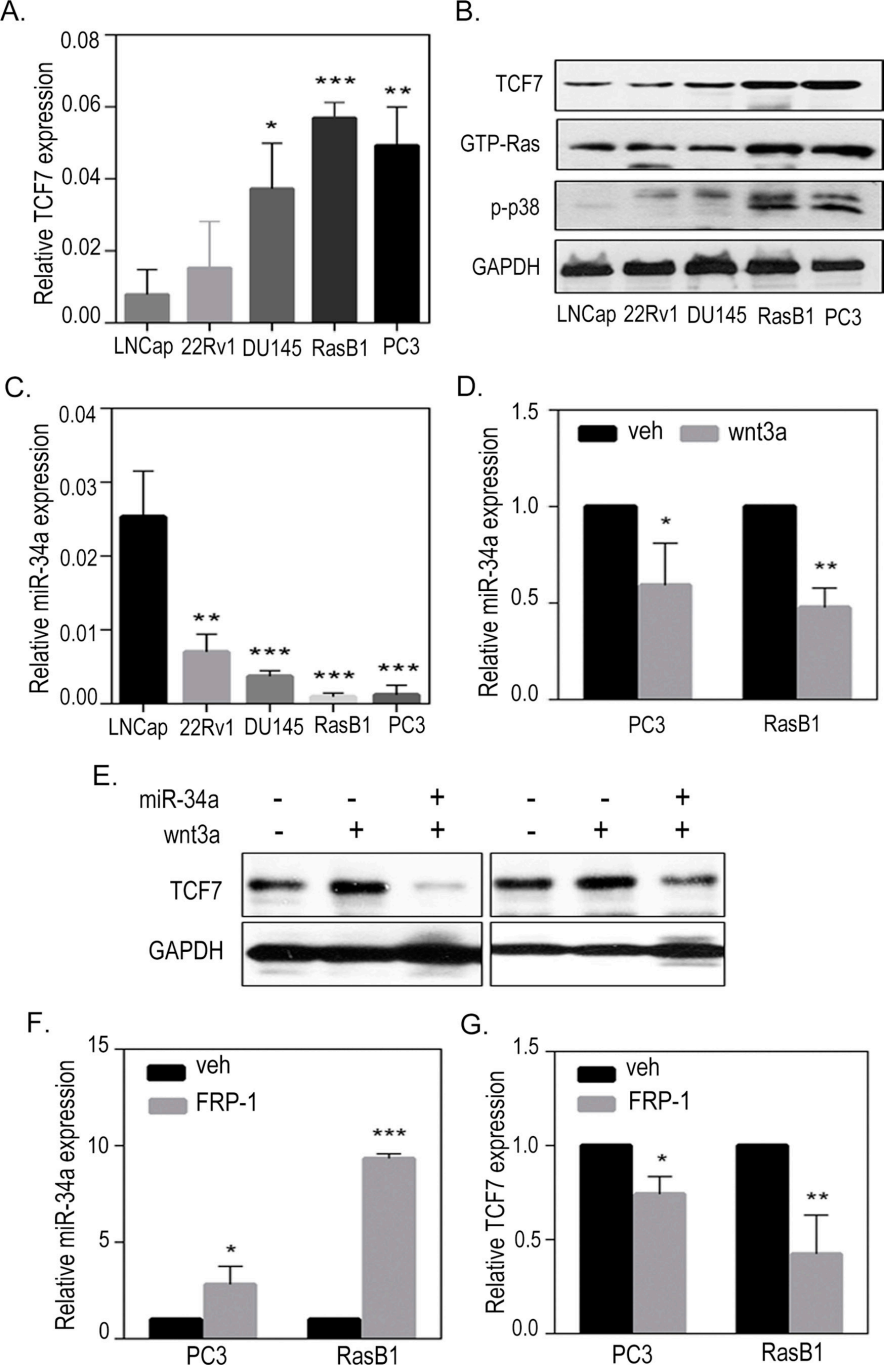
Activation of TCF7 in metastatic prostate cancer cells **(A)** qRT-PCR of *TCF7* level determined in LNCap, 22Rv1, DU145, RasB1, and PC3 cells. Relative mRNA expression was normalized to *GAPDH*. **(B)** Representative Western Blot analysis of TCF7, GTP-Ras, and phospho-p38 in LNCap, 22Rv1, DU145, RasB1, and PC3 cells. **(C)** qRT-PCR of miR-34a level determined in LNCap, 22Rv1, DU145, RasB1, and PC3 cells. **(D)** qRT-PCR analysis of miR-34a level in PC3 and RasB1 cells after being treated with wnt3a for 24 hours. Relative microRNA expression was normalized to *SNORD48*. *: vs. vehicle. **(E)** Representative Western Blot analysis of TCF7 in PC3 and RasB1 cells after being treated with wnt3a for 24 hours and transiently transfected with miR-34a. (F and G) qRT-PCR analysis of miR-34a **(F)** and TCF7 **(G)** levels in PC3 and RasB1 cells after being treated with FRP-1 for 24 hours. *: vs. vehicle. *p < 0.05. **p < 0.01, ***p < 0.001.

Having shown that Ras and WNT pathways have a synergistic role during prostatic tumorigenesis [35], we hypothesized that persistent Ras activation might explain the induction of the WNT signaling pathway via inducing the expression of WNT-related genes in advanced prostate cancer cells. TCF7, also known as TCF-1, is the most strongly up-regulated canonical WNT transcription factor, and has an established role in the nuclear response to WNT signaling, which enhances bone metastases [36, 37]. As expected, we found that the PC3 and RasB1 cells, which metastasized to the bone, had more RNA and protein expression levels of the canonical WNT response gene, TCF7, compared to the non-metastasizing cells (Figures 2A and 2B). Further confirming the correlation between Ras pathway dysregulation and WNT signaling in these cells, western blots for GTP-Ras and phospho-p38MAPK showed relatively increased Ras signaling and TCF7 level in PC3 and RasB1 cells (Figure 2B), suggesting a synergistic action of Ras and the WNT pathway in metastatic prostate cancer cells. Importantly, we also show a reduced expression level of miR-34a in these cells (Figure 2C), suggesting that TCF7 acts downstream of the Ras pathway and is involved in miR-34a regulation. Interestingly, a decreased level of miR-34a was detected in PC3 and RasB1 cells treated with the WNT ligand, wnt3a (Figure 2D), suggesting that the induced TCF7, via activated WNT signaling, is involved in miR-34a down-regulation. We confirmed that TCF7 protein level increased in wnt3a-treated PC3 and RasB1 cells; however, transient transfection with miR-34a precursor abolished the wnt3a-stimulated TCF7 activity (Figure 2E). In addition, wnt3a treatment induced the activation of WNT signaling and slightly decreased miR-34a and increased TCF7 expression in the non-metastatic cell lines LNCap and 22RV1 (Supplementary Figure S2A and S2B). In contrast, an increase in miR-34a expression (Figure 2F) and a decrease in endogenous TCF7 expression (Figure 2G) were observed in PC3 and RasB1 cells treated with the WNT inhibitor (FRP-1). Furthermore, the mean expression analysis of the clinical prostate database [6] showed reduced miR-34a and increased TCF7 expression in metastatic tumor samples compared to primary stage tumors and normal prostate tissues (Supplementary Figures S1C and S1D). These results indicate that stimulating the WNT pathway promotes TCF7, which is associated with miR-34a inactivation in metastatic prostate cancer cells.

### miR-34a directly binds to the 3′UTR of *TCF7* and regulates the stability of *TCF7* mRNA

**Figure 3:**
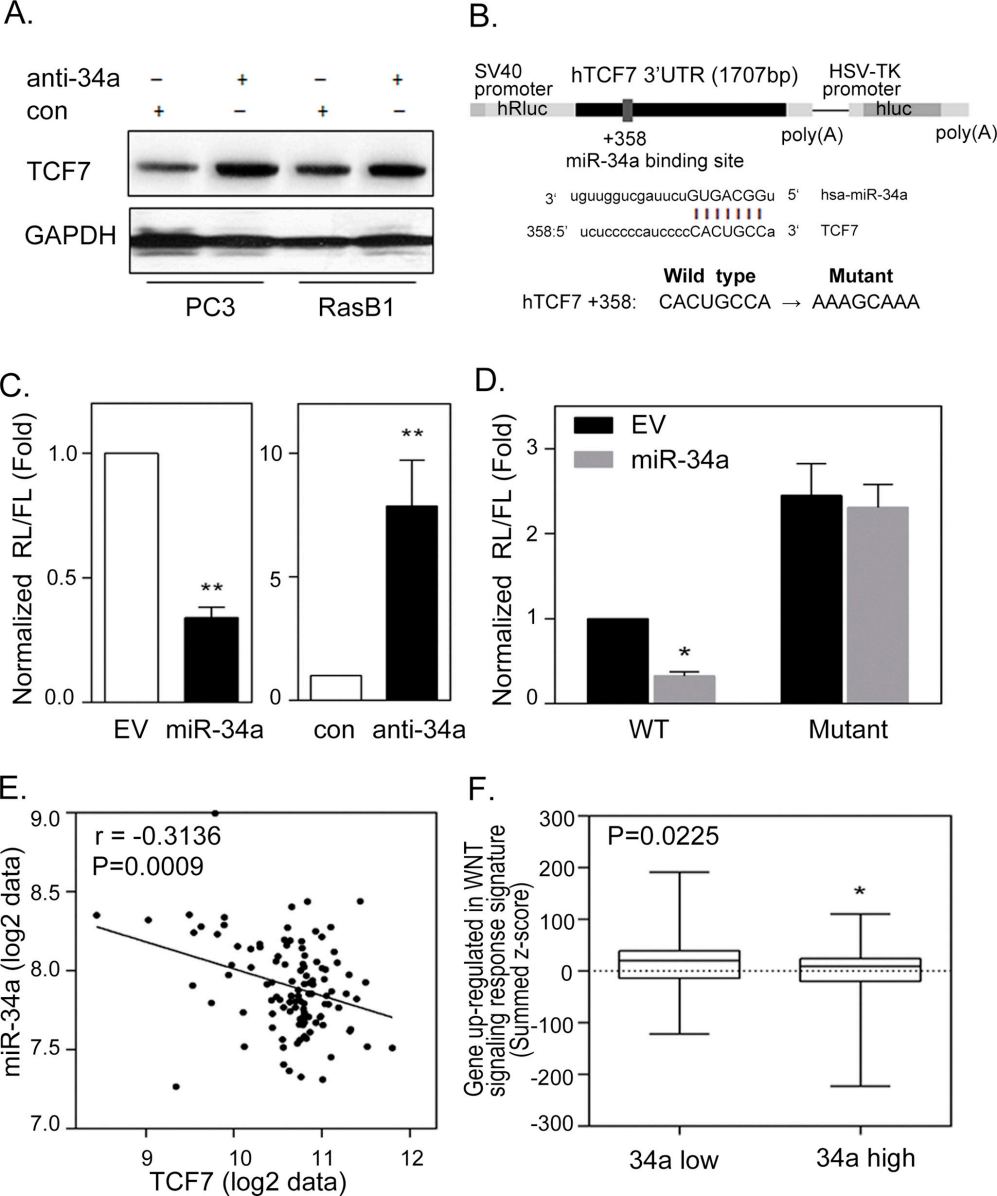
miR-34a regulates TCF7 expression by targeting *TCF7* 3′UTR in prostate cancer cells **(A)** Representative Western Blot analysis of TCF7 in PC3 and RasB1 cells following transient transfection with control or anti-miR-34a inhibitor. **(B)** Schematic of predicted miR-34a binding site and the introduced binding site mutant in full length 3′UTR reporter constructs of human *TCF7*. **(C)** The normalized reporter activity of the microRNA target reporter containing the full length human *TCF7* 3′UTR in RasB1 cells with transient expression of an empty vector (EV)/miR-34a precursor (left panel) or control (con)/anti-miR34a inhibitor (right panel). *: vs. EV or control. **(D)** The normalized reporter activity of *TCF7* 3′UTR containing wild type or mutated miR-34a target reporters in RasB1 cells with transient expression of an empty vector or miR-34a precursor. Renilla/luciferase activities were measured 48 hours after transfection. Data represent mean ± SEM of separate transfections, *n* = 3. *: vs. EV. *p < 0.05, **p < 0.01. **(E)** Pearson coefficient correlation of mean miR-34a to mean TCF7 mRNA expression in primary and metastatic prostate cancer samples (*n* = 111). Significance determined by Gaussian population (Pearson) and two-tailed test. **(F)** Mean summed z-scores for the miR-34a signature in the human prostate carcinoma dataset of the WNT signaling responsive genes set, showing that low miR-34a expressing patients have a high expression of WNT up-regulated responsive genes signatures.

It has been shown that miR-34a plays a critical role in prostate cancer where a decreased miR-34a expression is usually found in metastatic prostate cancer [24]. TCF7 was found to be expressed at high levels and associated with Ras signaling in prostate cancer [35]. We found that inhibiting miR-34a in PC3 as well as the Ras signaling-activated prostate cancer cell line, RasB1, was accompanied by an increase in TCF7, indicating that *TCF7* is a candidate target gene for miR-34a (Figure 3A). We hypothesize that activated Ras signaling, i.e. increased TCF7 expression, was caused by the inactivation of miR-34a. This is due to the loss of the transcriptional repressor function of miR-34a that targets the *TCF7* 3′UTR. To further investigate the binding sites of miR-34a, the homologous binding sites of miR-34a in the *TCF7* 3′UTR were analyzed (Figure 3B), and decreased luciferase activities were detected by reporter assay upon co-transfection with a miR-34a precursor (Figure 3C, left). Moreover, inhibition of miR-34a in RasB1 cells induced 3′UTR reporter activity of *TCF7* (Figure 3C, right). To further determine the relative contribution of miR-34a-dependent regulation, individual response elements and mutant miR-34a target site reporter constructs were prepared (Figure 3B). Reporter assays demonstrated a specific repressive role of miR-34a on the binding site (Figure 3D). These data suggest that miR-34a directly binds to the 3′UTR of *TCF7* and regulates the stability of *TCF7* mRNA. We explored the relevance of this finding to study the activation of the *TCF7* gene in a public human prostate cancer dataset [6]. To examine the inverse correlation between miR-34a and *TCF7* expression in prostate cancer progression, we analyzed the correlation of miR-34a and *TCF7* in primary and metastatic prostate cancer samples. The mean expression of *TCF7* was significantly inversely correlated to miR-34a expression by Pearson coefficient correction analysis (Figure 3E). Moreover, we observed a higher miR-34a expression in tumors that had lower WNT oncogenic response gene signatures in the human prostate cancer dataset (Figure 3F). These results are consistent with our observation linking miR-34a inactivation to a significantly increased *TCF7* expression, required in oncogenic WNT-activated prostate cancer.

### Clinical reversion of miR-34a induces TCF7 with poor prognosis in human prostate cancer patients

**Figure 4:**
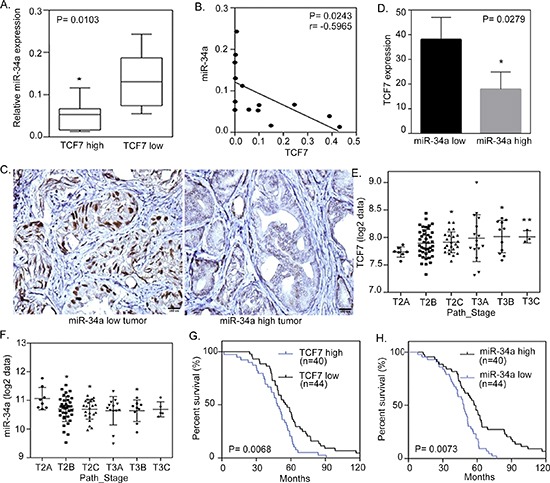
miR-34a negatively correlates with TCF7 expression in metastatic prostate cancer cells **(A)** qRT-PCR of miR-34a levels determined in 24 individuals with prostate cancer. qRT-PCR was used to classify tumors into two groups, low and high TCF7 expression. Significance determined by the Student's t test. **(B)** Negative correlation between mean miR-34a and mean TCF7 mRNA expression in metastatic prostate cancer samples (*n* = 14). Significance determined by Gaussian population (Pearson) and two-tailed test. **(C)** Representative immunohistochemical staining with antibodies specific for TCF7 are shown for 24 individual tissue sections from prostate cancers expressing high and low levels of miR-34a. Scale bars represent 100 μm. **(D)** Inverse correlation of TCF7 protein levels measured by IHC in tissues with low and high miR-34a expression. (**E** and **F**) TCF7 (E) and miR-34a (F) expressions in patient samples with the clinical stage in the prostate cancer samples collected and analyzed at Memorial Sloan-Kettering Cancer Center (MSKCC). *: vs. T2A. (G and H) Kaplan-Meier curve showing survival relative to TCF7 **(G)** and miR-34a **(H)** expression in prostate cancer samples collected and analyzed at MSKCC. The patient groups with high TCF7 and low miR-34a levels (blue line) have a lower survival compared to the groups with low TCF7 and high miR-34a levels (black line). Hazard ratios (Log rank) are 1.921 (TCF7) and 2.016 (miR-34a). X-axes show time in months and Y-axes show percentage survival (Log rank (Mantel-Cox) test *p* = 0.0068 (TCF7) and *p* = 0.0073 (miR-34a)).

To further study the inverse relation between miR-34a and its targets in human prostate tissue, we analyzed 24 independent prostate tumors, collected form Wan Fang Hospital, Taipei Medical University, Taiwan, which were divided into two groups of ‘low’ and ‘high’ *TCF7* expression, based on qRT-PCR analyses. An analysis of the variance confirmed that miR-34a was differentially expressed between the low and high expression level groups, where tissues with higher levels of *TCF7* expression had a lower miR-34a expression level (Figures 4A and 4B). Furthermore, immunohistochemistry analysis showed TCF7 overexpression in the nucleus of tumors that had distant metastasis and low expression levels of miR-34a (Figures 4C and 4D). In order to prove the correlation between TCF7 and miR-34a in cancer progression, we showed that there was an increased TCF7 and decreased miR-34a expression as the cancer stage increased (Figures 4E and 4F). To test the relationship between TCF7 and miR-34a expression with prostate cancer patient survival, Kaplan-meier analysis was performed and it showed that patients with a higher TCF7 and lower miR-34a expression had a lower survival rate (Figures 4G and 4H). Taken together, it appears that miR-34a regulates WNT signaling and acts as a tumor suppressor by down-regulating *TCF7* to prevent the progression of prostate cancer. These data further support the notion that a hyperactive WNT pathway reduces miR-34a expression, resulting in the activation of TCF7 and consequently leading to the promotion of malignant phenotypes of prostate cancer cells and poor clinical prognosis.

### miR-34a inhibits the development of a malignant phenotype of Ras-activated prostate cancer

**Figure 5:**
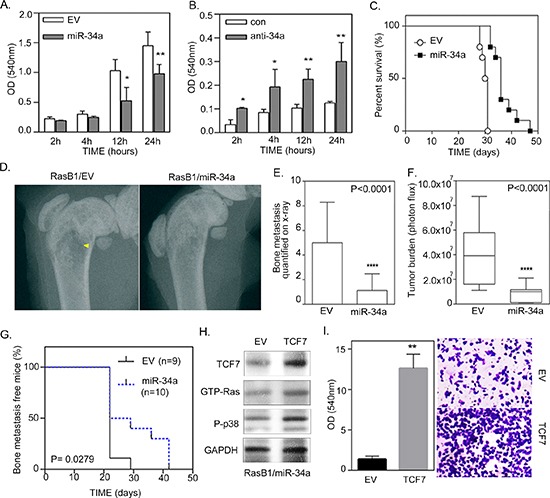
miR-34a regulates the invasion of Ras-activated prostate cancer cells *in vitro* and bone metastasis *in vivo* **(A)** Cellular invasion of RasB1 cells, infected with an empty vector (EV) or miR-34a precursor lentivirus, through Matrigel™-coated transwells for the indicated times, fixed and measured with ELISA reader at OD 540 nm. Data represent means ± SEM, *n* = 5. *: vs. EV. **(B)** Cellular invasion of DU145 cells transfected with 50 nM of control or anti-miR-34a inhibitor for the indicated times and measured with ELISA reader at OD 540 nm. Data represent means ± SEM, *n* = 5. *: vs. control inhibitor. *p < 0.05, **p < 0.01. **(C)** Intra-cardiac injections of mice with RasB1 cells expressing an empty vector (*n* = 9) or miR-34a precursor (*n* = 10) for the indicated times. Survival rate of tumor-bearing mice in each group. **(D)** Radiographic image of femurs from empty vector and miR-34a bearing mice as described in (C). Yellow arrow indicates bone destruction. **(E)** Bone metastasis scores per mouse in tumor bearing mice as described in (C). Mice were inoculated with RasB1 cells expressing empty vector or miR-34a. **(F)** Intra-cardiac injections of mice with RasB1 cells expressing an empty vector (*n* = 9) or miR-34a precursor (*n* = 10) for the indicated times. Representations, along with percent survival, via bioluminescence images at the time of the first appearance of long bone metastasis are shown. (G) Bioluminescence signal of bone metastasis per mouse for mice bearing tumor cells as described in (F) at week 4. ***p < 0.001 vs EV. **(H)** Representative Western Blot analysis in RasB1 cells harboring miR-34a precursor (RasB1/miR-34a), transfected with an empty vector (EV) or TCF7 expression vector. **(I)** Cellular invasion of RasB1/miR-34a cells, infected with an empty vector (EV) or miR-34a precursor, through Matrigel™-coated transwells for 24 hours, fixed and measured with ELISA reader at OD 540 nm.

In order to assess whether the down-regulation of miR-34a is necessary for KRAS-induced cellular transformation, we analyzed the functional effects of miR-34a on cell growth and invasion in Ras-activated prostate cancer cells. The *in vitro* cell growth assay confirmed the significant effect of miR-34a overexpression on growth rate reduction in RasB1 cells (Supplementary Figure S1E). Moreover, cell invasion was reduced when we overexpressed miR-34a precursor in RasB1 cells (Figure 5A). Importantly, inhibition of miR-34a induced *in vitro* cell growth and invasion in parental DU145 cells (Supplementary Figure S1F and Figure 5B) but not in LNCap and 22RV1 cells (Supplementary Figure S2C and S2D). Furthermore, miR-34a precursor was stably overexpressed in RasB1 cells, confirming the effect of miR-34a on the *in vivo* metastatic efficiency of the well-established Ras signaling-activated bone metastatic prostate cancer cells. Using the intra-cardiac injection mouse model, RasB1 cells, overexpressing the miR-34a precursor, showed a significant increase in survival rate (Figure 5C), and a significant decrease in bone metastasis (Figure 5D to 5G) compared to an empty vector. Having demonstrated that ectopic miR-34a precursor expression decreased *TCF7* expression (Figure 2E) and reduced RasB1 cell invasiveness (Figure 5A), we sought to know whether the reconstitution of *TCF7* levels in miR-34a expressing cells would be sufficient to restore their invasive activity. The rescue experiment was performed by ectopic expression of TCF7 in miR-34a precursor-transfected RasB1 cells. Interestingly, inducing TCF7 expression increases GTP-Ras and p-p38 expression (Figure 5H), implying that TCF7 is downstream of miR-34a. As shown in Figure 5I, ectopic expression of TCF7 significantly reconstitutes invasiveness in pre-miR-34a-expressing cells. Taken together, these data suggest that *TCF7* is an important miR-34a target that augments invasiveness in Ras-activated prostate cancer cells. These data show that miR-34a suppresses a variety of metastatic properties, as well as growth rate, in advanced Ras signaling-activated prostate cancer cells.

### miR-34a overexpression contributes to the induction of apoptosis in Ras-activated prostate cancer cells

**Figure 6:**
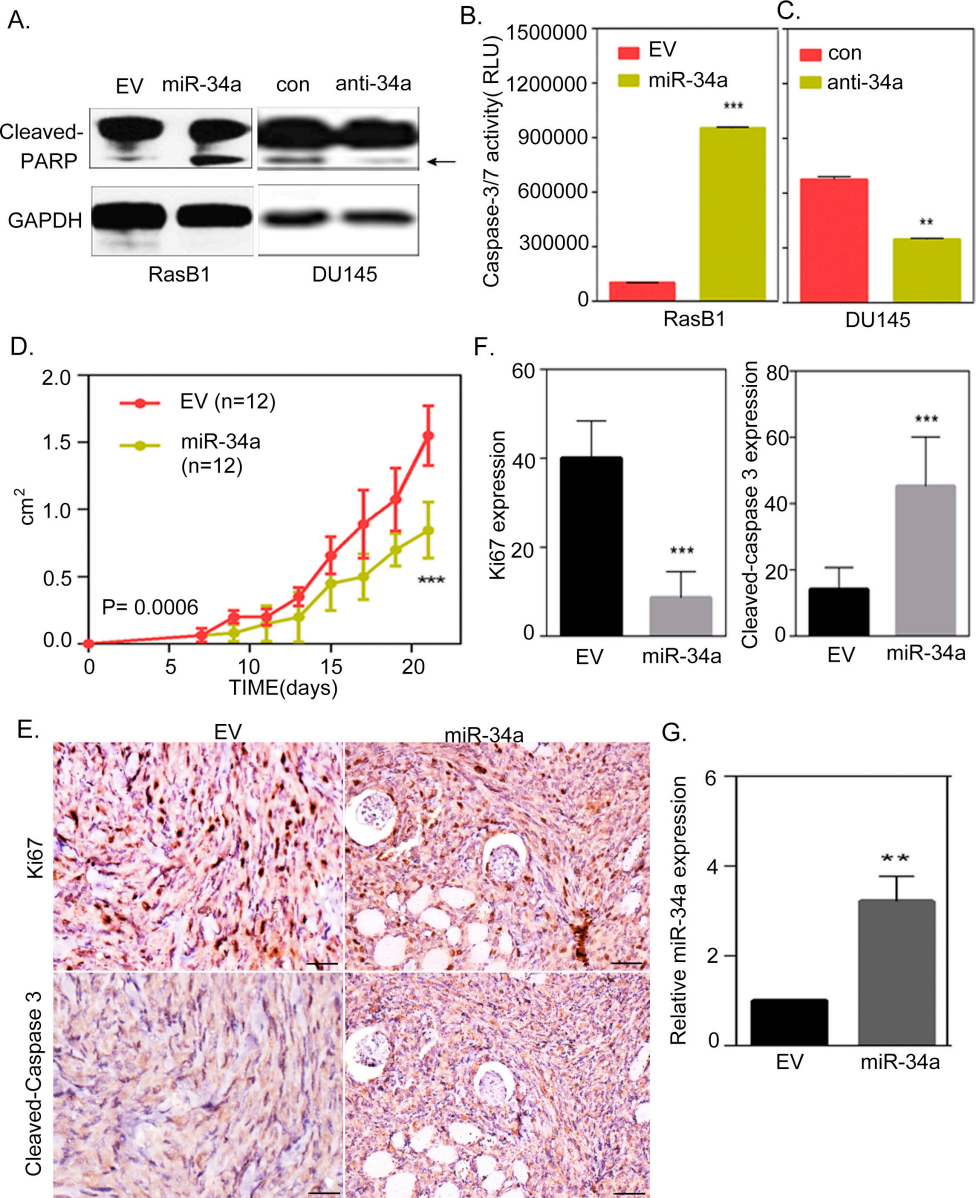
miR-34a overexpression contributes to the induction of apoptosis in Ras-activated prostate cancer cells **(A)** Representative Western Blot analysis of cleaved-PARP in RasB1 cells transfected with an empty vector (EV) or miR-34a precursor, and in DU145 cells transfected with control or anti-miR-34a inhibitor. **(B)** RasB1 cells were transfected with miR-34a precursor, apoptosis was assessed by measuring caspase-3/7 activity with relative luciferase unit (RLU). Data represent means ± SEM, *n* = 3. *: vs. EV. **(C)** Parental DU145 cells were transfected with anti-miR-34a inhibitor, and apoptosis was assessed by measuring caspase-3/7 activity with relative luciferase unit. Data represent means ± SEM, *n* = 3. *: vs. control. **(D)** Growth curve of xenograft tumors in nude mice (*n* = 12) injected with RasB1 cells stably infected with precursors of miR-34a or an empty vector as a control. **(E)** Representative immuno-histochemical analysis and antibodies specific for Ki67 and cleaved-caspase 3 are shown for consecutive tissue sections of RasB1 cells expressing an empty vector or miR-34a precursor. Scale bars represent 100 μm. **(F)** Inverse correlations of Ki67 and cleaved-caspase 3 protein level measured by IHC in tissue sections from (E). **(G)** Confirmed up-regulation of miR-34a in the xenograft tumors by qRT-PCR. qRT-PCR analysis of miR-34a measured in RNA isolated from subcutaneous tumors formed by genetically-altered RasB1 cells as indicated in (D). *: vs. EV. **p < 0.01, ***p < 0.001.

Anti-apoptosis is the most clinically important feature of prostate cancer [29], and it has been shown that the activation of Ras signaling is significantly associated with this cell survival technique in prostate cancer [25]. *KRAS*-mutant cancer cells are unresponsive to apoptosis because oncogenic Ras signaling, through the RAF pathway, involves an apoptotic response that is mediated by p53 [38]. Importantly, miR-34a is an important factor of the p53 network where miR-34a is a direct transcriptional target of p53 [23, 39, 40]. The miR-34 family has been shown to have anti-proliferative and apoptotic roles [22], so we sought to study the effect of miR-34a expression and its contribution to apoptosis in Ras signaling-activated prostate cancer cells. We assessed the relevance of miR-34a induced apoptosis and found that the cells harboring the miR-34a precursor had an increased cleaved-PARP expression in RasB1 cells; however, inhibiting miR-34a expression reduced PARP cleavage in DU145 cells (Figure 6A). Furthermore, miR-34a overexpression induced caspase-3/7 activity in RasB1 cells compared to cells that were transfected with the empty plasmid vector (Figure 6B). On the other hand, the parental DU145 cells had a decreased caspase-3/7 activity when transfected with anti-miR-34a compared to control anti-miR (Figure 6C). These results suggest that miR-34a overexpression induces apoptosis signaling in Ras-activated prostate cancer cells. Further confirming the relevance of *in vivo* miR-34a-induced apoptosis in Ras signaling-activated cells, we found that the overexpression of miR-34a in RasB1 cells resulted in a marked inhibition of tumor growth in nude mice (Figure 6D). In addition to that, we observed a reduction in Ki67- and an increase in cleaved-caspase 3-positive cells in xenograft tumors overexpressing miR-34a, respectively (Figures 6E and 6F). We confirmed the up-regulation of miR-34a in the xenograft tumors by qRT-PCR (Figure 6G). Taken together, RasB1 cell growth is inhibited through the up-regulation of miR-34a, which in turn affects anti-apoptotic signaling by directly regulating the anti-apoptotic signaling-related genes.

### Activated Ras signaling induces an anti-apoptotic pathway that is regulated by miR-34a

**Figure 7:**
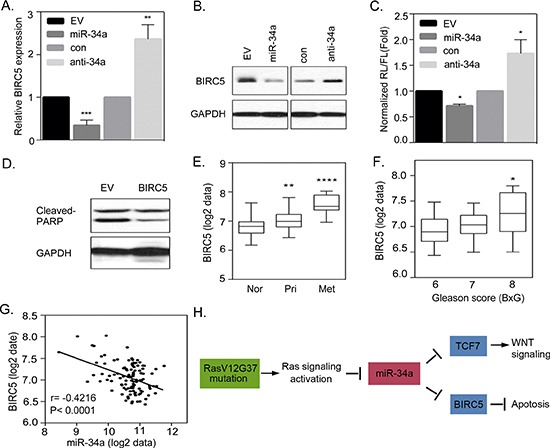
Activated Ras signaling induces an anti-apoptotic pathway that is regulated by miR-34a (**A** and **B**) qRT-PCR and Western Blot analysis of BIRC5 expression in RasB1 cells transfected with an empty vector (EV)/miR-34a precursor or control/anti-miR-34a inhibitor. Relative mRNA expression was normalized to *GAPDH*. Data represent means ± SEM, *n* = 3. *: vs. EV or control. **(C)** The normalized reporter activity of *BIRC5* 3′UTR containing miR-34a target reporter in RasB1 cells with transient expression of an empty vector (EV)/miR-34a precursor or control (con)/anti-miR34a inhibitor. Renilla/luciferase activities were measured 48 hours after transfection. Data represent mean ± SEM of separate transfections, *n* = 3. *: vs. EV or control. *p < 0.05. **(D)** Representative Western Blot analysis of cleaved-PARP in RasB1 harboring miR-34a cells transiently transfected with an empty vector or BIRC5 expression vector. **(E)** Mean mRNA expression of BIRC5 in human normal (*n* = 28), primary (*n* = 98), and metastatic (*n* = 13) prostate samples from the Taylor dataset. Significance determined by one-way ANOVA. *: vs. normal. **(F)** Mean BIRC5 expression in clinical Gleason score (BxG) in the prostate cancer samples from the Taylor dataset. *: vs. BxG6. **(G)** Pearson correlation coefficient analysis of mean BIRC5 to mean miR-34a expression in primary and metastasis prostate samples (*n* = 111). Significance determined by Gaussian population (Pearson) and two-tailed test. **(H)** A proposed model describing the interactions of miR-34a with TCF7 and BIRC5, leading to the activation of WNT signaling and apoptosis in RAS-activated prostate cancer.

When testing for the presence of miR-34a, which is associated with induced apoptosis in Ras signaling-activated cells, our results showed that RasB1 cells that are overexpressing miR-34a, show dramatically increased apoptosis (Figure 6). We hypothesize that miR-34a overexpression induces apoptosis in RasB1 cells by down-regulating anti-apoptotic genes. To validate that miR-34a is a regulator of apoptosis via an apoptotic signaling pathway, we assayed a number of predicted miR-34a targets that are associated with the established anti-apoptotic signaling pathway and examined whether the expression of the expected miR-34a targets could be regulated by miR-34a. The results showed that the levels of the candidate miR-34a target, the anti-apoptotic protein, BIRC5, was markedly reduced in the presence of the miR-34a precursor, and an increase in BIRC5 expression was observed in the presence of anti-miR-34a in RasB1 cells (Figure 7A and 7B). Furthermore, we sought to know whether the predicted miR-34a binding sites in the 3′UTR of *BIRC5* provide specificity, by monitoring the luciferase activities of the reporter constructs. As shown in Figure 7C, we showed that the exogenously expressed miR-34a precursor represses the luciferase activities and that the knockdown of miR-34a increases the signals, supporting a physical interaction between miR-34a and *BIRC5* 3′UTR.

*BIRC5* has been previously found to be the target gene of miR-34a in breast and colorectal cancers [41, 42], and our data further confirmed this relation in prostate cancer. To further confirm that the pro-apoptotic effect of miR-34a is mediated through the suppression of BIRC5 in Ras signaling-activated prostate cancer cells, we overexpressed *BIRC5* in RasB1 cells harboring the miR-34a precursor. The results showed that the apoptotic effect of miR-34a was reversible by the overexpression of BIRC5 with decreased cleaved-PARP expression (Figure 7D), suggesting that *BIRC5* is directly regulated by miR-34a and has a critical role in promoting anti-apoptotic signaling in Ras signaling-activated prostatic cells. We explored the relevance of this finding by studying the expression of BIRC5 in a public human prostate cancer dataset [6]. The mean expression intensity analysis of the clinical prostate database showed an increased expression of *BIRC5* in metastatic tumor samples (Figure 7E) and tumors with higher clinical Gleason scores (Figure 7F). To examine the relation between miR-34a and BIRC5 expression in prostate cancer progression, we employed a Pearson coefficient correction analysis and found that the mean expression of *BIRC5* was significantly inversely correlated to miR-34a expression (Figure 7G). In addition to that, we observed a correlation between Ras activation, BIRC5, and TCF7 expression in prostate cancer samples. This indicats that tissues from non-tumor prostate tissues, primary and metastatic stage prostate cancer tissues dataset, with down-regulated KRAS signatures, have more TCF7 and BIRC5 expression (Supplementary Figures S3A and S3B). These results are consistent with our observation linking Ras activation to a significantly increased TCF7 and BIRC5 expression in prostate cancer. Furthermore, knocking down TCF7 did not significantly attenuate the effect of anti-miR-34-transfected RasB1 cells in reducing BIRC5 expression (Supplementary Figure S4A). Moreover, we have tested for invasiveness by ectopically expressing BIRC5 in cells expressing miR-34a, and our data showed that over-expression of BIRC5 did not increase the invasive capability of cells harboring miR-34a (Supplementary Figure S4B). In conclusion, our study demonstrates the regulatory mechanisms by which miR-34a targets the WNT cascade and anti-apoptotic signaling: there is a direct link between the loss of miR-34a, activation of the canonical WNT signaling and anti-apoptotic pathways (Figure 7H).

## DISSCUSSION

An abnormal WNT/β-catenin pathway is found in several types of cancer, including colorectal, liver and prostate cancer [43]. β-Catenin activates T-Cell factor/lymphoid enhancer factor-1 (TCF/LEF-1) transcriptional activity and up-regulates genes such as MYC, MMP7 and vascular endothelial growth factor [43]. In prostate cancer, Ras mutations are relatively uncommon [44], however, the Ras/MAPK pathway has also been implicated in prostate cancer development and progression, where it was found to be activated in 90% of metastatic samples [5]. Ras effector pathways are emerging as prime potential therapeutic targets for treating androgen-independent prostate cancer [45]. The combinatorial oncogenic activations of Ras and WNT signaling drive the rapid progression of prostate tumorigenesis into invasive carcinoma [35]. In this paper, our results highlight the importance of the Ras and WNT cascades during prostate cancer progression, characterized by a miR-down-regulated canonical WNT signaling gene, *TCF7*, in androgen-independent prostate cancer cells. We showed that miR-34a could be reduced by Ras signaling and that it functions as a negative regulator of WNT signaling by directly targeting the 3′UTR of *TCF7*. Reduction of miR-34a leads to an oncogenic effect on cell growth and invasion in Ras signaling-activated prostate cancer cells.

We found that two bone metastatic prostate cancer cell lines (PC3 and a RasG37-mutated RasB1 cell line) [34] have induced TCF7 and Ras signaling-related genes expression (Figure 2A and 2B). Furthermore, our results showed that miR-34a expression was down-regulated when PC3 and RasB1 cells were treated with the canonical WNT ligand, wnt3a (Figure 2D). Transient transfection with the precursor miR-34a abrogated the stimulation of TCF7 activity mediated by wnt3a (Figure 2E). This highlighted miR-34a as a potential, previously unidentified, link between wnt3a and TCF7-mediated oncogenesis. Conversely, FRP-1, which down-regulates WNT signaling, up-regulated miR-34a expression, and TCF7 activity was subsequently reduced (Figure 2F and 2G). Following the silencing of miR-34a in bone metastatic PC3 and RasB1 cells, we observed an increase in TCF7 expression levels (Figure 3A), suggesting that reduced levels of miR-34a are required for the up-regulation of TCF7. By screening miRs that are expected to regulate TCF7, we identified that miR-34a directly interacted with *TCF7* 3′UTR and regulated its 3′UTR reporter activity (Figure 3B to 3D). In addition, our clinical data showed that miR-34a expression is significantly higher in prostate cancer patients with a low TCF7 expression (Figure 3E and 3F, Figure 4A and 4B), and significantly lower in tumors expressing a high nuclear level of TCF7 (Figure 4C). This indicated again the importance of miR-34a expression in regulating TCF7 levels and the potential clinical relevance of this study. Moreover, a direct link between miR-34a activity and the Ras signaling pathway was shown by p53-transactivated miR-34a, where the Ras pathway reduces the expression of p53, which in turn suppresses the post-transcriptional activity of miR-34a and promotes apoptosis in breast cancer [39]. In accordance with our experimental findings, ectopically expressed miR-34a inhibits the *in vivo* bone metastatic properties and growth rate of Ras-activated prostate cancer cells (Figure 5 and 6). Our data indicates that miR-34a suppresses the development of a malignant phenotype in advanced Ras signaling-activated prostate cancer.

There have been previous hints of a connection between miR-34a and apoptosis. For instance, the anti-apoptotic proteins *BCL2* and *ALDH2* are known to be implicated in a number of cancers and to contain putative miR-34a binding sites within their 3′UTRs [22, 46]. Although we showed an inverse correlation between TCF7 and miR-34a, which could be mediated by Ras signaling activation, the negative effect of miR-34a on cell proliferation could not be explained only by the down-regulation of TCF7 via miR-34a. In accordance with this hypothesis, we showed an inhibitory effect of miR-34a on Ras-activated prostate cancer cell proliferation (Figure 6). These findings strongly indicate that miR-34a also regulates genes, other than TCF7, involved in regulating cell proliferation. Interestingly, the search for candidate miR-34a targets revealed that genes that positively regulate apoptotic signaling also contain the seed region, for miR-34a interaction, in their 3′UTRs. An important anti-apoptotic protein, *BIRC5* (also known as Survivin) was found to contain a miR-34a binding region, and which was down- and up-regulated when using a miR-34a precursor and inhibitor in Ras-activated prostate cancer cells, respectively (Figure 7A to 7C).

Survivin is commonly associated with an enhanced proliferative index [47], reduced level of apoptosis [48], resistance to chemotherapy [49], and increased rate of tumor recurrence [50]. Survivin was also studied in larynx squamous cell carcinoma, which had reduced levels of miR-34a, and was found to be correlated with cell survival, metastasis, differentiation and clinical stage [41]. Following transfection with a miR-34a mimic, Survivin expression was down-regulated without a decrease in mRNA expression, along with a decrease in proliferation and an arrest at the G0/G1 phase in larynx squamous cell carcinoma cell lines [41]. Furthermore, miR-34a was found to down-regulate Survivin via the E2F3 transcription factor, thereby inhibiting proliferation, migration, colony formation and survival of human head and neck squamous cell carcinoma cell lines, and inhibit tumor angiogenesis in SCID mice by blocking VEGF and inhibiting endothelial function [51]. Our study demonstrated that Survivin is a unique miR-34a target in Ras signaling-activated prostate cancer cells and was found to promote the anti-apoptotic pathway in prostate cancer cells harboring miR-34a (Figure 7D). This observation is consistent with our hypothesis that links miR-34a inactivation to a significantly increased BIRC5 expression, which is required in oncogenic Ras-activated prostate cancer.

Dysregulation of the apoptotic pathway is one of the factors that are associated with chemoresistance in prostate cancer [52]. Here, we have elucidated one of the mechanisms by which Ras signaling activation down-regulates cellular apoptosis and induces cell proliferation. We have conducted the first functional study to evaluate the role of miR-34a in Ras signaling-activated prostate cancer and highlighted its therapeutic potential for the treatment of synergistic activations of Ras and WNT signaling cascades. Our study contributes to a better understanding of prostate cancer progression where re-expression of miR-34a was found to dramatically inhibit prostate cancer cell proliferation and to regulate the expression of anti-apoptotic proteins. This might help clinical oncologists to plan a more rational and efficient therapeutic strategy to deal with the problems of Ras signaling targeted-therapy resistant patients. Therefore, in Ras signaling-activated prostate cancers, miR-34a replacement could stop the stimulation of cell growth by WNT signaling and may be an adjunct to current anti-WNT signaling therapies.

## MATERIALS AND METHODS

### Reagents and constructs

Wnt3a was from R&D (R&D Systems, MN) and recombinant FRP-1 was from GeneTex (GeneTex, CA). BD Matrigel™ was purchased from BD Biosciences (BD Biosciences, CA) for the invasion assay. miR precursors (empty vector and miR-34a precursor) and anti-miR inhibitors (control and anti-miR-34a) were from GeneCopoeia (GeneCopoeia, MD). For lentiviral-mediated over-expression of miR-34a, we used the miR-34a stem-loop expression vector from GeneCopoeia (GeneCopoeia, MD), based on FIV packaging kit manual. Human BIRC5 and TCF7 full length expression vector and empty vector were from GenScript (GenScript, NJ). Human *TCF7* full length 3′UTR reporter was constructed using the psiHECKTM-2 vector (Promega, WI). The microRNA binding site mutation was made using the Site-Directed Mutagenesis System kit (Invitrogen, CA). All primers used for these constructs are listed in Supplementary Table S2. All constructs were verified by DNA sequence analysis.

### Cell culture

PC3 and RasB1 cell lines were cultured in RPMI 1640 media supplemented with 10% fetal calf serum (FCS). The RasB1 cell line was modified from the human prostate cancer cell line, DU145, by stably expressing a RasG37 mutation construct [33]. RasB1 cell line was isolated from the bone metastatic site following DU145/RasG37 orthotropic injection [34]. Transient transfections were carried out using Lipofectamine RNAiMAX (Invitrogen, CA). The dosage of wnt3a and WNT inhibitor was: wnt3a (100 ng/ml), and FRP-1 (0.5 μg/ml) in serum free condition.

### Invasion assay

Cellular invasion of RasB1 cells, infected with an empty vector (EV) or miR-34a precursor lentivirus, was assessed on Matrigel™-coated transwells. We seeded 1 × 10^6^ cells per well and added RPMI 1640 media supplemented with 10% FCS in both chambers. After 2, 4, 12, and 24 hours of incubation at 37°C, membranes were fixed and stained with 0.5% crystal violet. After removing the non-invading cells from the top of the membrane, the invading cells were dissolved in 50% ethanol containing 0.1M sodium citrate and quantified by an ELISA reader at OD 540 nm. Five individual wells were counted for each time point. Invading DU145 cells were transiently transfected with 50 nM control or anti-miR-34a inhibitor.

### *In vitro* growth assay

Cells were plated at a density of 2 × 10^3^ cells per well. Inducing the expression of miR-34a precursor and anti-miR-34a inhibitor of RasB1 and DU145 cells were performed as described in the part on invasion assay. Each day, one plate was stained with 0.5% crystal violet fixative solution for 15 minutes, rinsed in distilled water, and allowed to air dry. At the end of the experiment, crystal violet was dissolved by adding 100 μl of 50% ethanol containing 0.1M sodium citrate to each well, and the absorbance was quantified, at a wavelength of OD 540 nm, on a plate reader.

### Real-time RT-PCR

Total RNA was isolated using mirVana PARIS RNA isolation system (Ambion, TX). Reverse transcription into cDNA and PCR were performed as previously described [53]. All reactions were normalized to human *GAPDH* and were run in triplicates using primers listed in Supplementary Table S3. MicroRNA reverse transcription and PCR reactions were performed using TaqMan MicroRNA Assay kits (Applied Biosystems, CA). All values were normalized to human *SNORD48* endogenous control and were run in triplicates (Applied Biosystems, CA). The 24 clinical samples of patients with independent prostate tumors used in qRT-PCR analyses were collected form Wan Fang Hospital, Taipei Medical University, Taiwan. RNA was extracted from dissected tissues containing more than 70% tumor cell content.

### MicroRNA luciferase assay

Cells were transfected with 1μg of human *TCF7* 3′UTR reporter and 1μg of a precursor miR, encoding an empty vector or miR-34a precursor. The psiCHECK-2 vectors contain both firefly and Renilla luciferase reporters. Cell extracts were prepared 24 hours after wnt3a (100 ng/ml) or FRP-1 (0.5 μg/ml) treatment and the luciferase and Renilla activities were measured using the Dual Luciferase Reporter Assay System (Promega, WI). Renilla luciferase activities were calculated as mean ± SEM after normalization to firefly luciferase activities. Three independent experiments were done in triplicates. The microRNA binding sites on human *TCF7* 3′UTR were determined using the Computational Biology Center, Memorial Sloan Kettering Cancer Center website (microRNA.org) and Bioinformatics and Research Computing, Whitehead Institute for Biomedical Research (TargetScan.org).

### Western blot analysis

Cell lysis was performed with RIPA buffer containing complete protease inhibitors (Roche, CA), phosphatase inhibitors (Roche, CA), 25 mM β-glycerophosphate, 10 mM sodium fluoride and 1 mM sodium vanadate as previously described [53]. Primary antibodies were incubated overnight at 4°C using the dilutions listed in Supplementary Table S4.

### Immunohistochemistry (IHC) staining

The 24 clinical samples of independent primary prostate tumors were collected from Wan Fang Hospital, Taipei Medical University, Taiwan. Immunohistochemistry (IHC) was performed using antibodies as indicated in Supplementary Table S5. Unstained sections were deparaffinized and rehydrated. Antigen retrieval was performed using Target Antigen Retrieval Solution (DAKO, CA) followed by a 10-minute autoclave. For IHC, endogenous peroxidase was blocked using a 3% hydrogen peroxide solution. All sections were blocked with Cyto Q Background Buster Reagent (Innovex BioSciences, CA). Primary antibodies were incubated overnight at 4°C in Antibody Diluent with Background Reducing Components (DAKO, CA). Secondary antibody, 1:250 HRP labeled anti mouse/rabbit (Vector laboratories, CA), incubation was performed at room temperature for 30 minutes and bound peroxidase was detected using the ABC Peroxidase Kit (Vector laboratories, CA) and DAB (DAKO, CA). All IHC slides were counterstained with hematoxylin. For histomorphometric analysis of tissue sections, microscopic images were collected under 200X magnification using an Axioplan microscopy system (Zeiss, Thornwood, New York). ImageJ software (NIH, USA) was used to quantify the expression of TCF7 in different prostate tumor sections, where the immuno-ratio of DAB/nuclear area was calculated in each section and the average of 5 random sections was plotted.

### Clinical outcome and correlation analyses using human data sets

We used the mRNA expression data from a public human prostate cancer dataset [6]. The study was conducted under MSKCC Institutional Review Board approval on 28 normal, 151 primary, and 19 metastatic samples. The expression data (and resulting z-scores) were log2 normalized. Additionally, microRNA expression was determined for 98 primary tumors, 13 metastatic tumors, and 28 matched normal samples with Agilent microRNA V2 arrays. WNT signaling (Broad Institute), KRAS signaling [54], and prostate metastasis [55] responsive gene signatures were used to determine correlations with miR-34a or BIRC5 levels. Gene sets were scored by summing the expression z-scores per tumor within the cohort. Tumors were mean stratified by miR-34a or BIRC5 expression and the mean z-scores were determined for each group. Gene set enrichment analysis (GSEA) software from the Broad Institute [56] was used to assess the significance of prostate cancer metastasis responsive gene signature by false discovery rate (FDR). Concordant overexpression of all genes in a signature (compared with the mean expression of all genes) leads to a high positive score and indicates the presence of the signature in the tumor. The Kaplan-Meier curve shows the survival rate relative to log2 normalized TCF7 or miR-34a expression.

### Animal studies

Animal work was performed in accordance with a protocol approved by the NIH Animal Care and Use Committee. To analyze metastasis, five-week old male nude mice (NCI, Frederick) were subjected to intra-cardiac injections with 1 × 10^5^ tumor cells and bioluminescent imaging (BLI) was performed as previously described [53]. For survival studies, mice were euthanized when one of the following situations applied: 10% loss of body weight, paralysis, or head tilting. Bone metastases were evaluated on magnified (3X) radiographs taken with Faxitron MX-20. Each bone metastasis was scored based on the following criteria. 0: no metastasis, 1: bone lesion covers less than ¼ of the bone width, 2: bone lesion involves ¼ to ½ of bone width, 3: bone lesion across ½ to ¾ of bone width, 4: bone lesion is more than ¾ of bone width. The bone metastasis score for each mouse represents the sum of scores of all bone lesions from four limbs. To analyze tumorigenesis, five-week old male nude mice were subcutaneously injected with 1 × 10^6^ tumor cells in 50% Matrigel™.

### Statistical analysis

*In vivo* animal results and clinical outcome analysis were expressed as plots showing the median and box boundaries extending between 25th to 75th percentiles, with whiskers down to the minimum and up to the maximum value. All *in vitro* data were presented as means ± S.E.M. Statistical calculations were performed with GraphPad Prism (GraphPad Software, Inc.) analysis tools. Differences between individual groups were analyzed by one way or two way ANOVA test. Bonferroni's post test was used for comparisons among 3 or more groups. Log-rank test was used for survival curve analysis. *P*-value less than 0.05 was considered statistically significant.

## SUPPLEMENTARY FIGURES AND TABLES


