# Supplementary material for: MicroRNA-34a regulates WNT/TCF7 signaling and inhibits bone metastasis in Ras-activated prostate cancer
Source: Oncotarget. 2014 Nov 25;6(1):441–57. doi: 10.18632/oncotarget.2690 (PMC4381606; doi:10.18632/oncotarget.2690)
Supplement: Supplementary file 1 [file oncotarget-06-441-s001.pdf]

SUPPLEMENTARY FIGURES AND TABLES

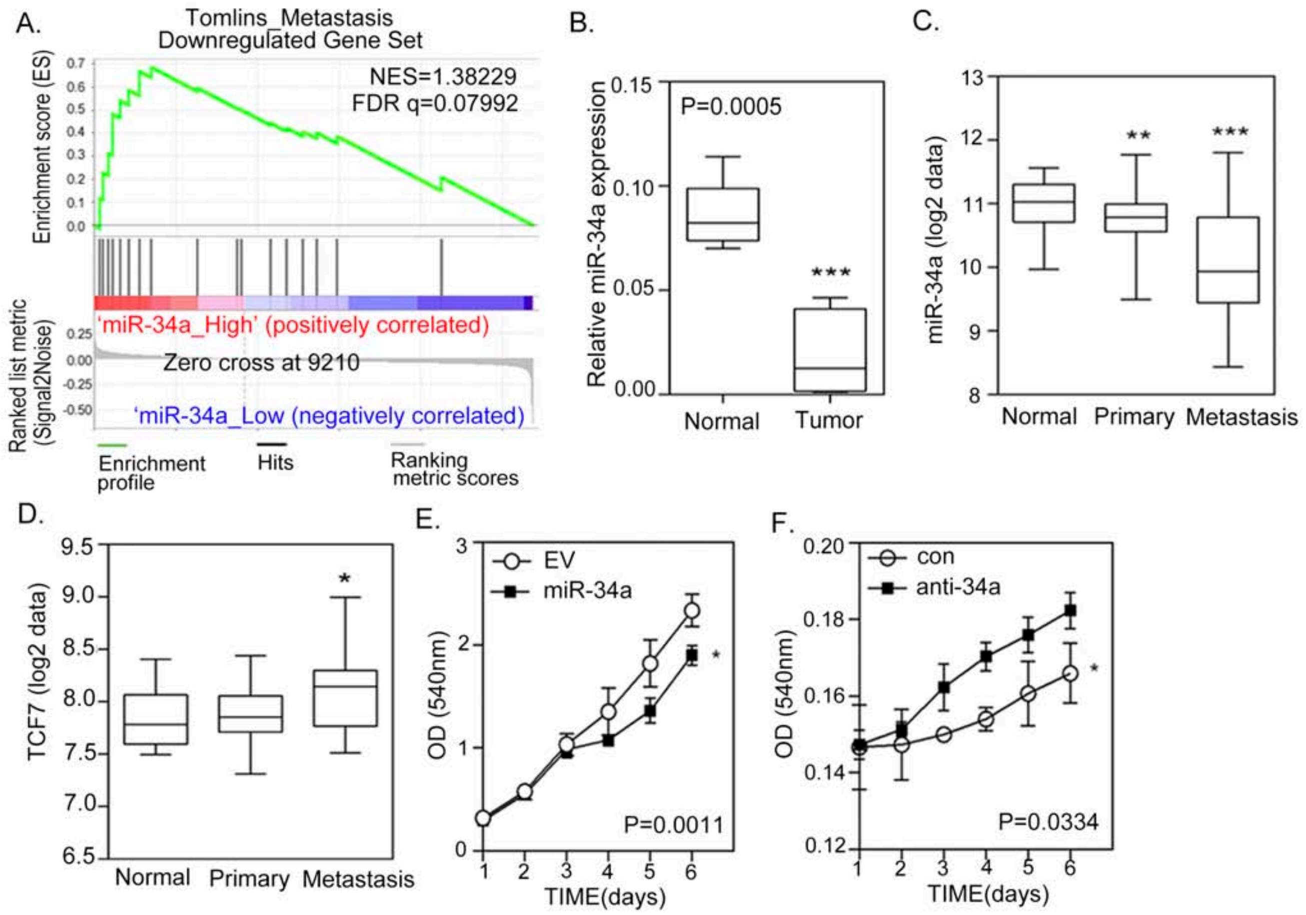

Supplementary Figure S1: Effects of miR-34a expression in prostate cancer cells. **(A)** Gene set enrichment analysis (GSEA) showing enrichment of Taylor prostate cancer dataset of prostate cancer metastasis down-regulation responsive genes in prostate cancer highly expressing miR-34a. False discovery rate (FDR), normalized enrichment score (NES). **(B** and **C)** Mean miR-34a (B) and mRNA expression of TCF7 (C) in human normal (n = 28), primary (n = 98), and metastatic (n = 13) prostate samples from the Taylor dataset. Significance determined by one-way ANOVA. *: vs. primary. **(D)** Representative data of the *in vitro* growth curve of RasB1 cells expressing an empty vector (EV) or miR-34a precursor for the indicated times and measured with ELISA reader at OD 540 nm. Data represent means ± SEM, n = 6. *: vs. EV. **(E)** Cellular growth curve of DU145 cells transfected with 50 nM of control or anti-miR-34a inhibitor for the indicated times and measured with ELISA reader at OD 540 nm. Data represent means ± SEM, n = 5. *: vs. control inhibitor. *$p < 0.05$, **$p < 0.01$, ***$p < 0.001$.

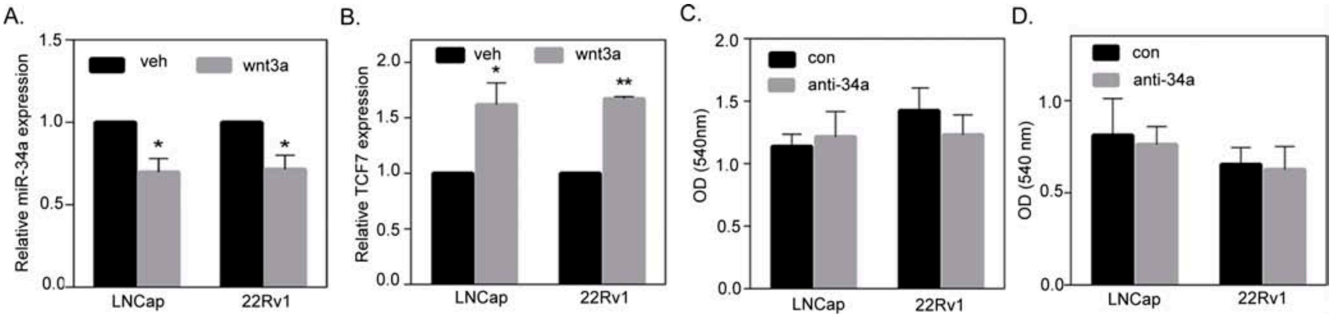

Supplementary Figure S2: The effect of miR-34a in non-metastatic prostate cancer cells. **(A** and **B)** qRT-PCR analysis of miR-34a (A) and TCF7 (B) levels in LNCap and 22Rv1 cells after being treated with wnt3a for 24 hours. Relative miRNA and mRNA expressions were normalized to *SNORD48* and *GAPDH*, respectively. *: vs. vehicle. **(C** and **D)** Cellular migration (C) and invasion (D) of LNCap and 22Rv1 cells, transfected with 50 nM of control or anti-miR-34a inhibitor, through transwells (C) or Matrigel™-coated transwells (D) for 24 hours, fixed and measured with ELISA reader at OD 540 nm.

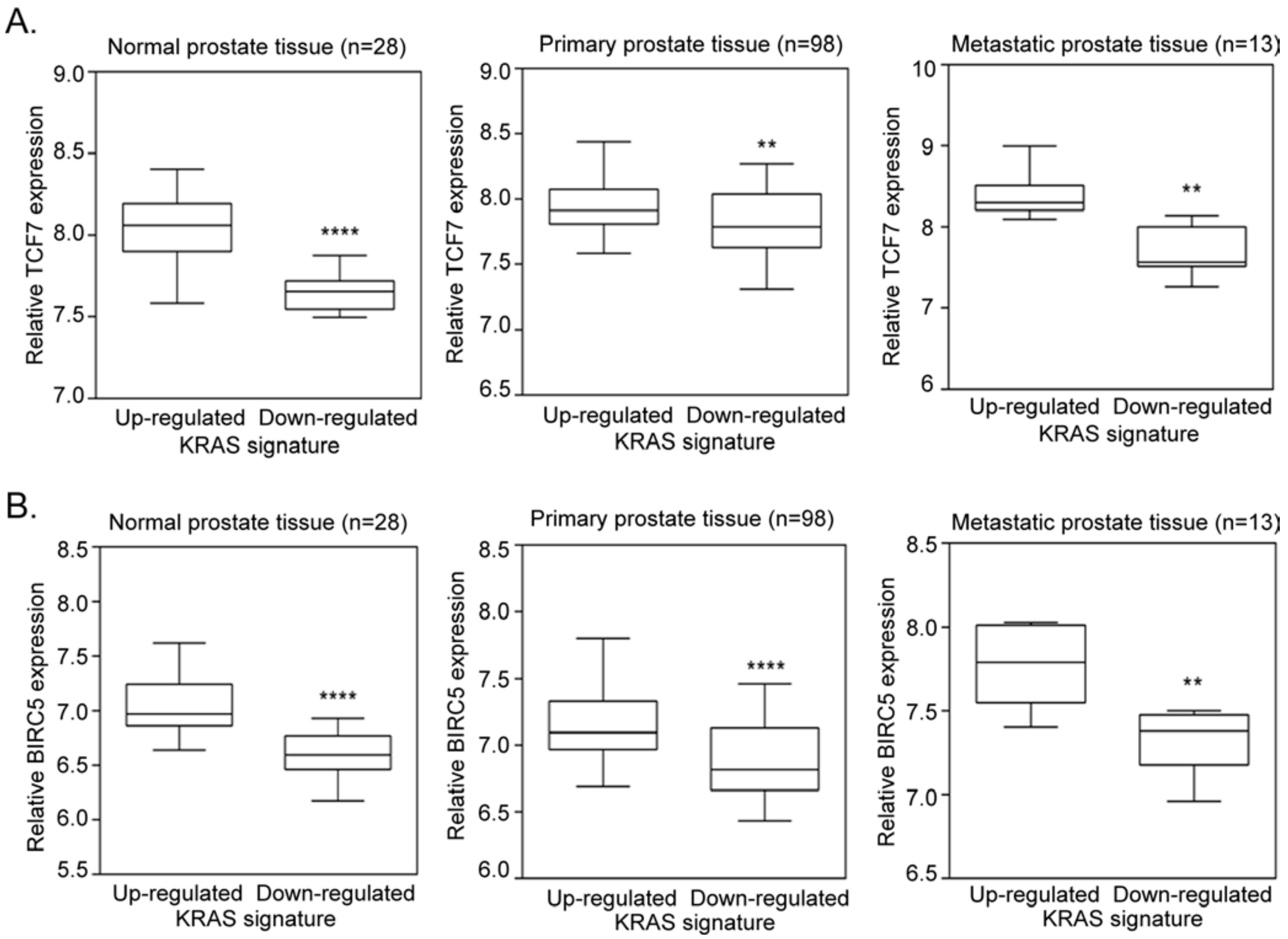

Supplementary Figure S3: A correlation of Ras activation, miR-34a, TCF7, and BIRC5 expressions in prostate cancer samples. **(A)** Summed z-scores for TCF7 expression in the human prostate carcinomas dataset, segregated into up- and down- regulated KRAS signatures in tissues of normal (left), primary (middle), and metastatic (right) stage prostate cancer. **(B)** Summed z-scores for the BIRC5 expression in the human prostate carcinomas dataset. segregated into up- and down- regulated KRAS signatures in tissues of normal (left), primary (middle), and metastatic (right) stage prostate cancer.

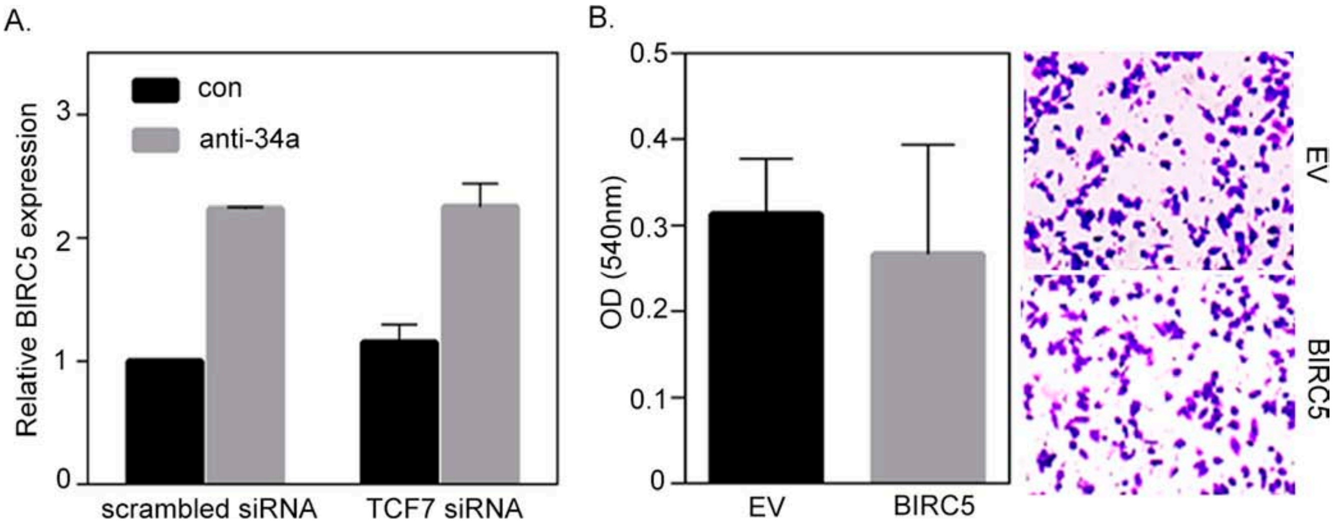

Supplementary Figure S4: miR-34a mediates BIRC5 expression independently of TCF7. **(A)** qRT-PCR analysis of BIRC5 level in RasB1 cells after being treated with scrambled siRNA or TCF7 siRNA for 24 hours. **(B)** Cellular invasion of RasB1/miR-34a cells, infected with an empty vector (EV) or BIRC5 expression vector, through Matrigel™-coated transwells for 24 hours, fixed and measured with ELISA reader at OD 540 nm.

Supplementary Table S1. The different cell lines used in this study

Cell line	LNCap	22Rv1	DU145	PC3	V12	G37	RasB1
Derived cells	-	-	-	-	DU145	DU145	DU145
KRAS mutation status	none	none	none	none	RasV12	RasV12G37	RasV12G37
Metastatic potential	none	none	moderate	high	high	high	high

Supplementary Table S2. The primer sequence of 3′UTR and promoter reporter constructs

**Human TCF7 3′UTR reporter constructs primer sequence**	
TCF7/psi-2 3′UTR (XhoI) F	ctcgag CCTACCCCCTGAAAGTGACA
TCF7/psi-2 3′UTR (PmeI) R	gtttaaac CAGGCTTTGAAAAACAAACC
TCF7 34a-1M F	CTACCCATCTCCCCCATCCCCAAAGCAAACACCCTCCCC
TCF7 34a-1M R	CGATGGAAGAGATGGGTAGAGGGGGTAGGGG
**Human BIRC5 3′UTR reporter constructs primer sequence**	
BIRC5/psi-2 3′UTR (XhoI) F	atcgctcgagACCTGAAAGCTTCCTCGACA
BIRC5/psi-2 3′UTR (PmeI) R	attcgtttaaacTGTGAGTTACTCTTTCCACATGG

Supplementary Table S3. The primer sequence of qRT-PCR

Gene	5′-3′
TCF7 F	CTGCCATCAACCAGATCCT
TCF7 R	GCTCATAGTACTTGGCCTGCT
BIRC5 F	AAGGACCACCGCATCTCTAC
BIRC5 R	CAAGTCTGGCTCGTTCTCAG
GAPDH F	CCAGTAGAGGCAGGGATGAT
GAPDH R	CTTTCATTGTCTTTTCCGCC

Supplementary Table S4. The antibody information from WB

Primary antibody	Source	Dilution	Secondary antibody	Source	Dilution
TCF7	Cell Signaling (#2203)	1/1000	anti rabbit IgG	Thermo Scientific	1/2000
PARP	Cell Signaling (#9532)	1/1000	anti rabbit IgG	Thermo Scientific	1/2000
GTP-Ras	Abcam (ab96548)	1/1000	anti rabbit IgG	Thermo Scientific	1/2000
P-p38 MAPK	Cell Signaling (#9219)	1/1000	anti rabbit IgG	Thermo Scientific	1/2000
BIRC5	Cell Signaling (#2808)	1/1000	anti rabbit IgG	Thermo Scientific	1/2000
Gapdh	Novus (NB300-221)	1/4000	anti mouse IgG	Thermo Scientific	1/2000

Supplementary Table S5. The antibody information from IHC

Primary antibody	Source	Dilution	Antigen Retrieval	Secondary antibody	Source	Dilution
TCF7	Cell Signaling (#2203)	1/100	Autoclave	HRP anti-Rabbit	DAKO	1/400
Ki67	Abcam (ab13847)	1/300	Autoclave	HRP anti-Rabbit	DAKO	1/400
Cleaved-caspase 3	Abcam (ab15580)	1/300	Autoclave	HRP anti-Rabbit	DAKO	1/400
